# Supplementary material for: Accounting for endogenous effects in decision-making with a non-linear diffusion decision model
Source: Sci Rep. 2023 Apr 18;13:6323. doi: 10.1038/s41598-023-32841-9 (PMC10113207; doi:10.1038/s41598-023-32841-9)
Supplement: Supplementary file 1 — Supplementary Information. [file 41598_2023_32841_MOESM1_ESM.pdf]

1 Comparing the Ornstein-Uhlenbeck and the nl-DDM model

Other non-linear models of decision-making exist, among which the Ornstein-Uhlenbeck (OU) model[1]. This model takes the form:

$$dx(t) = (\lambda x(t) + \mu)dt + N(t)$$

We note that, similar to the nl-DDM, the variation depends on the current state of the decision $x(t)$. μ corresponds to the effect of the stimulus, identical to the effect of the drift in the DDM. λ represents the effect of the state of the participant on the accumulation process. To represent an accumulation process for a two-alternative forced-choice task, accumulation boundaries have to be fitted in addition to these parameters. Due to their similarity in interpretation and differences in formalism, a comparison between the nl-DDM and the OU model was performed. To compare its fitting performance to these of the nl-DDM, we performed a 5-fold cross-validation in order to compare the fitting performances and to account for the complexity of both models. Indeed, the functional form of the OU drift is simpler than the nl-DDM equation but implements additional absorbing boundaries, which are a highly non-linear phenomenon, adding complexity to the model. Then, we fitted both models on the lexical classification dataset. The nl-DDM was defined using a pair of stable fixed-points $\pm a$ per instruction condition, one time scale k, the middle of the starting point distribution x_0 and its half-width s_z, a non-decision time T_{nd}, and fours unstable fixed-points z_1, z_2, z_3, z_{NW}, corresponding to frequent, rare, very rare, and non-existent word stimuli respectively. For the OU model, λ, $\mu_1, \mu_2, \mu_3, \mu_{NW}$ (one per stimulus type), one boundary per instruction condition, a non-decision time, the middle of the starting point distribution x_0 and its half-width s_z were fitted. For both models, the noise scale was set to $\sigma = 0.3$. The models were therefore defined using the same number of parameters. Note that, as opposed to the main analysis presented in the article, this analysis did not consider the effect of time passing to reduce the computation cost in anticipation of the computational cost of cross-validation.

Data was then split into 5 folds, ensuring that each fold contained the same number of trials in each block, each instruction condition, and each stimulus type to ensure the uniformity of data presented. Both the nl-DDM and OU models were fitted to 4 folds and the fitting performance of the fitted model was tested on the remaining fold, so that each fold was used as the test set once. We then compare the train and the test Negative Log-Likelihood of the two models pairwise for each participant and each fold.

We observed that the test scores of the nl-DDM were significantly better than those of the OU model (Shapiro-Wilk test: $W = 0.412, p < 0.001$; one-sided paired Wilcoxon signed-rank test, $W(80) = 349, p < 0.001, d = 0.785$), meaning that the nl-DDM generalizes better than the OU, indicating a simpler model form. Although this result might seem surprising given that the nl-DDM is defined by a third-order differential equation and the OU with a linear form, it is noteworthy that the OU necessitates the definition of additional absorbing decision boundaries, which is mathematically ill-defined because of its highly non-linear nature. Consequently, its fitting is numerically challenging.

2 Correlation analysis on empirical data

The goal of the correlation analysis presented in the main paper was to underline links between the DDM and nl-DDM parameters. In this part, we are interested in knowing whether these relations are also found empirically.

We computed the Pearson's correlation coefficients of the nl-DDM parameters over all conditions and participants, using only the multi-sensory classification dataset for simplicity, i.e., over $N = 50$ observations. This allows supporting the observations we have noted in the formalism part. Indeed, since fewer parameters were fitted in this case than for the lexical classification dataset, the comparison becomes more straightforward. From the 25 participants, we obtained 50 fits per model type by duplicating for each stimulus type the boundaries and time constant terms, hence separating the stimulus types and obtaining 25×2 fits per model type.

The results of this analysis are presented Figure S1 and S2. The correlations within nl-DDM parameters observed on simulated data are found again. We observe that some of the correlations across nl-DDM and DDM parameters are modified due to DDM parameters not being fixed, as opposed to the analysis on simulated data presented in the main article.

3 Behavioral analysis of the lexical classification dataset[5]

The tables of ANOVAs on the behavior held by participants on the lexical classification task are given Tables S1, S2 and S3. We assessed the effects of word type, instruction and time of the experiment on both RT and accuracy by performing repeated-measures ANOVAs.

The RT varied significantly with word type ($F(3,45) = 36.329, p < 0.001$, Supplementary Table S1), instruction ($F(1,15) = 16.541, p = 0.001$), and time ($F(1,15) = 55.260, p < 0.001$), with a significant interaction effect of word type and instruction ($F(3,45) = 3.305, p = 0.021$), word type and time ($F(3,45) = 82.976, p < 0.001$), and word type, time and instruction ($F(3,45) = 9.579, p < 0.001$). Post-hoc comparisons revealed that non-existent words resulted in significantly shorter RTs than

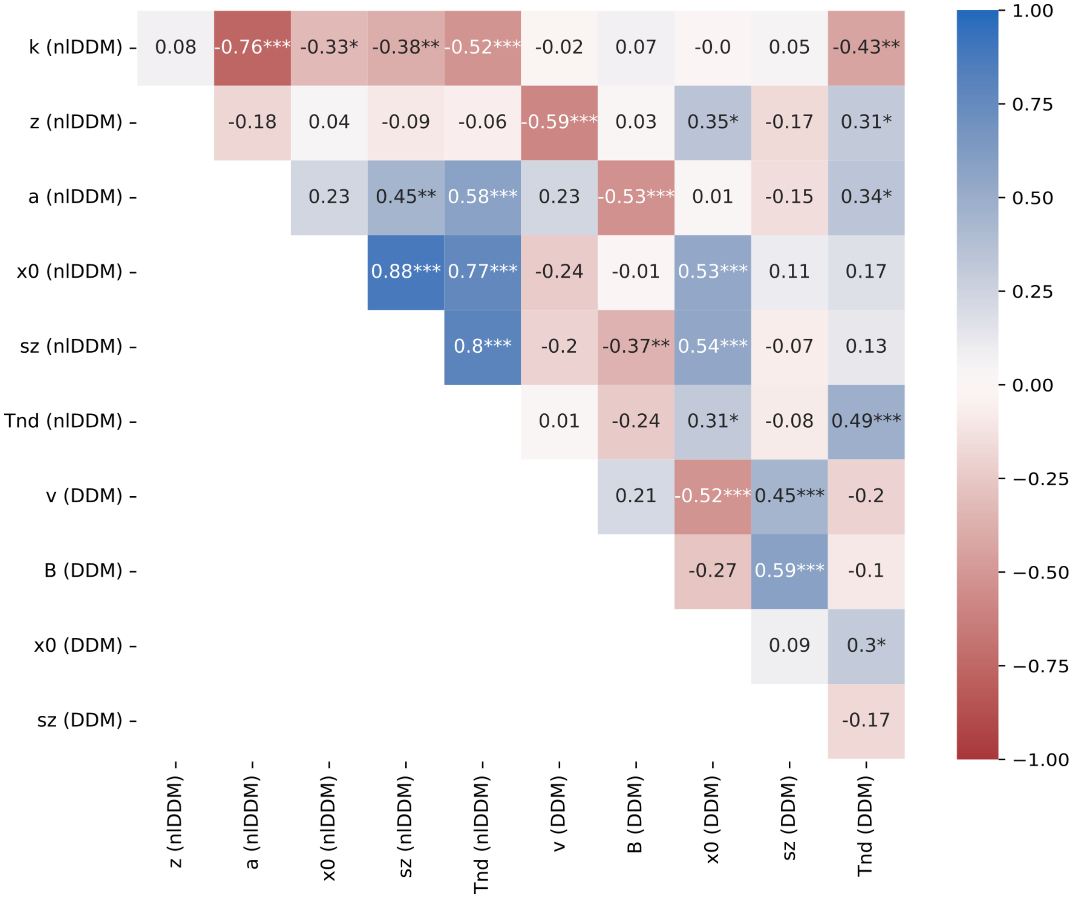

Figure S1. Pearson correlation coefficients between DDM and nl-DDM parameters, nl-DDM and DDM parameters fitted over the multi-sensory dataset. This figure was obtained using the matplotlib (3.5.2)[2]-based Python library seaborn (0.11.2)[3] (see https://matplotlib.org/ and https://seaborn.pydata.org/)

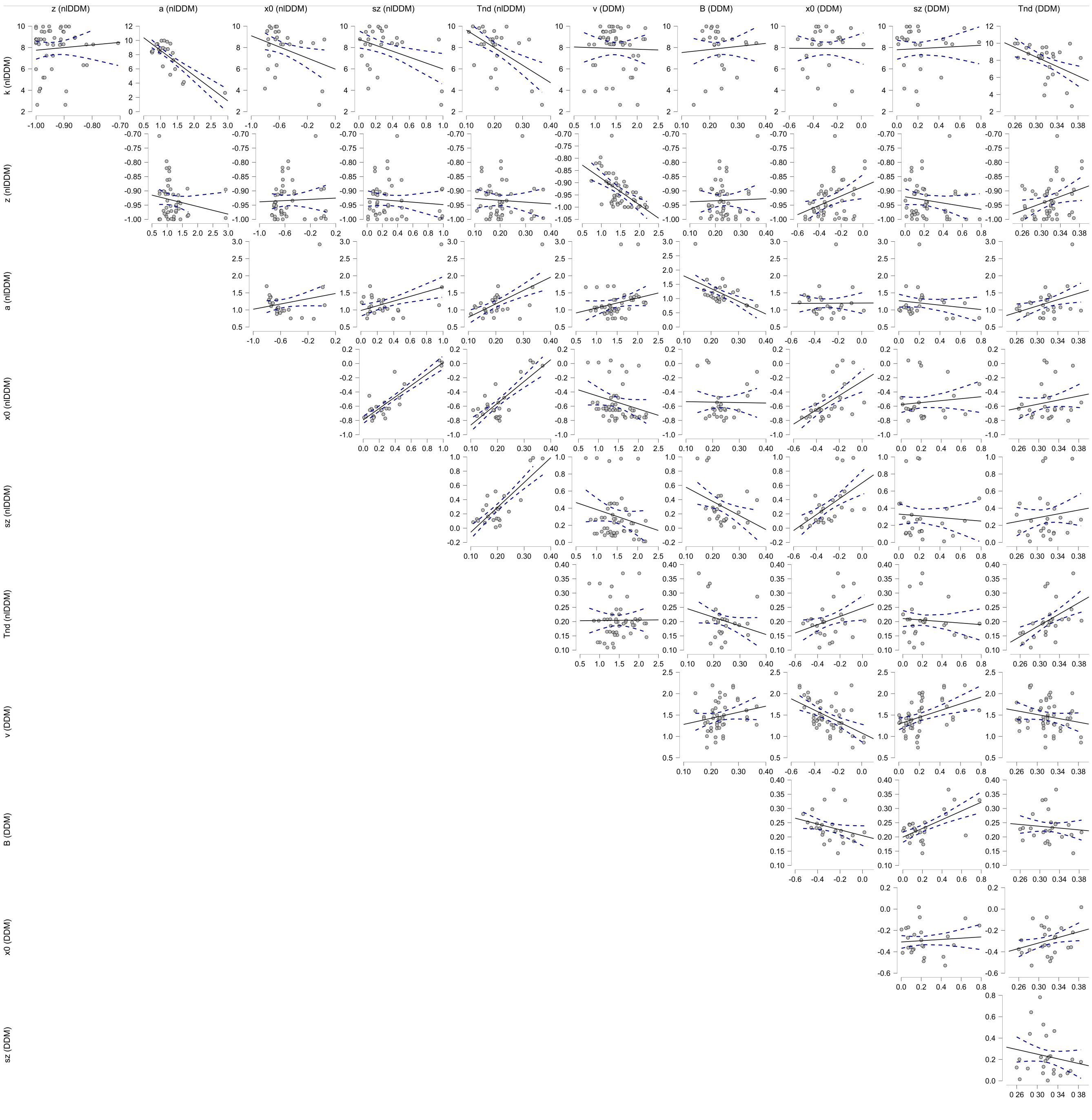

Figure S2. Correlation plots of DDM and nl-DDM parameters, computer from the fitting over the multi-sensory classification dataset. The correlations were computed as Pearson's correlation coefficient.The blue dashed lines represent the 95% confidence intervals for the regression. This figure has been generated using JASP (0.16.0.0)[4](see https://jasp-stats.org/).

any other word type, frequent words shorter RTs than rare or very rare words, and rare words yield prompter responses than very rare words (Supplementary Table S3).As expected, participants also responded significantly faster in the speed condition compared to the accuracy condition ($t(15) = 4.067, p_{Holm} = 0.001$). Interestingly, participants responded faster in late relative to early trials ($t(15) = 7.434, p_{Holm} < 0.001$).

The accuracy was also significantly impacted by word type ($F(3,45) = 179.581, p < 0.001$, Supplementary Table S2), instruction ($F(1,15) = 23.863, p < 0.001$), time ($F(1,15) = 102.297, p < 0.001$), and the interaction between word type and time ($F(3,45) = 54.412, p < 0.001$), and word type, time and instruction ($F(3,45) = 3.163, p = 0.034$). Post-hoc analyses revealed that both frequent and non-existent words were responded to more accurately than both rare and very rare words (frequent-rare: $t(15) = 18.567, p_{Holm} < 0.001$, frequent-very rare: $t(15) = 11.197, p_{Holm} < 0.001$, non-existent-rare: $t(15) = 19.874, p_{Holm} < 0.001$, non-existent-very rare: $t(15) = 12.504, p_{Holm} < 0.001$). Participants also responded significantly more accurately to rare words compared to very rare words ($t(15) = 7.370, p_{Holm} < 0.001$). Accuracy trials were more accurate on average than speed trials ($t(15) = 4.885, p_{Holm} < 0.001$). Early trials were more accurate than late trials ($t(15) = 10.114, p_{Holm} < 0.001$).

From these results, it is noteworthy that the time of the experiment significantly impacts behavior, and seems to imply modifications in the speed-accuracy trade-off strategy. Note also that there is no interaction effect between time and instruction.

Table S1. ANOVA on Response Times of the lexical classification task

Cases	Sum of Squares	df	Mean Square	F	p
Word type	2.998	3	0.999	36.329	$< .001$
Residuals	1.238	45	0.028		
Time	0.073	1	0.073	55.260	$< .001$
Residuals	0.020	15	0.001		
Instruction	0.044	1	0.044	16.541	0.001
Residuals	0.040	15	0.003		
Word type * Time	0.427	3	0.142	82.976	$< .001$
Residuals	0.077	45	0.002		
Word type * Instruction	0.021	3	0.007	3.223	0.031
Residuals	0.099	45	0.002		
Time * Instruction	$7.895e-4$	1	$7.895e-4$	0.882	0.363
Residuals	0.013	15	$8.950e-4$		
Word type * Time * Instruction	0.023	3	0.008	9.579	$< .001$
Residuals	0.035	45	$7.843e-4$		

Table S2. ANOVA on Mean accuracy of the lexical classification task

Cases	Sum of Squares	df	Mean Square	F	p
Word type	6.591	3	2.197	179.581	$< .001$
Residuals	0.551	45	0.012		
Time	0.057	1	0.057	102.297	$< .001$
Residuals	0.008	15	$5.549e-4$		
Instruction	0.108	1	0.108	23.863	$< .001$
Residuals	0.068	15	0.005		
Word type * Time	0.230	3	0.077	54.412	$< .001$
Residuals	0.063	45	0.001		
Word type * Instruction	0.001	3	$4.380e-4$	0.337	0.799
Residuals	0.059	45	0.001		
Time * Instruction	0.001	1	0.001	2.195	0.159
Residuals	0.010	15	$6.618e-4$		
Word type * Time * Instruction	0.008	3	0.003	3.163	0.034
Residuals	0.039	45	$8.717e-4$		

Table S3. Post Hoc Comparisons of Word type effect on response times in the lexical classification dataset

		Mean Difference	SE	t	p_{holm}
non-existent	frequent	−0.061	0.029	−2.097	0.042
	rare	−0.276	0.029	−9.410	$< .001$
	very rare	−0.193	0.029	−6.587	$< .001$
frequent	rare	−0.214	0.029	−7.314	$< .001$
	very rare	−0.132	0.029	−4.491	$< .001$
rare	very rare	0.083	0.029	2.823	0.014

References

1. Busemeyer, J. R. & Townsend, J. T. Decision field theory: A dynamic-cognitive approach to decision making in an uncertain environment. *Psychol. Rev.* **100**, 432–459, DOI: 10.1037/0033-295X.100.3.432 (1993).
2. Hunter, J. D. Matplotlib: A 2d graphics environment. *Comput. Sci. & Eng.* **9**, 90–95, DOI: 10.1109/MCSE.2007.55 (2007).
3. Waskom, M. L. seaborn: statistical data visualization. *J. Open Source Softw.* **6**, 3021, DOI: 10.21105/joss.03021 (2021).
4. JASP Team. JASP (Version 0.16.4)[Computer software] (2022).
5. Wagenmakers, E.-J., Ratcliff, R., Gomez, P. & McKoon, G. A diffusion model account of criterion shifts in the lexical decision task. *J. Mem. Lang.* **58**, 140–159, DOI: 10.1016/j.jml.2007.04.006 (2008).
